# Aggressive Primary Thyroid Mucoepidermoid Carcinoma with Extensive Pulmonary Involvement

**DOI:** 10.3390/biomedicines12020285

**Published:** 2024-01-26

**Authors:** Marius Mitrache, Dana Terzea, Anca Sirbu, Simona Fica

**Affiliations:** 1 Endocrinology Department, Carol Davila University of Medicine and Pharmacy, 020021 Bucharest, Romania; lucian.mitrache@gmail.com (M.M.); danaterzea@gmail.com (D.T.); simonafica55@gmail.com (S.F.); 2 Endocrinology Department, Elias Emergency University Hospital, 011461 Bucharest, Romania; 3 Oncoteam Diagnostic, 010719 Bucharest, Romania

**Keywords:** mucoepidermoid cancer of thyroid, aggressive behavior, rare cancer

## Abstract

Mucoepidermoid carcinomas (MECs) represent the most common malignant neoplasms of the salivary glands, but they have also been described in other unusual sites. Primary MECs originating in the thyroid gland are exceedingly rare, accounting for less than 0.5% of thyroid tumors. Owing to their low to medium grade, they are usually associated with an indolent evolution and a good long-term prognosis, generally being managed surgically based on the extent of the disease. However, this does not always apply, as primary thyroid MECs may present as metastatic or locally advanced diseases. While several treatment options have been explored in such cases, no consensus currently exists on their optimal treatment plan, and they should be managed in a multidisciplinary fashion. We report the case of a 67-year-old patient with primary MEC of the thyroid, which behaved aggressively, with extensive pulmonary metastasis, ultimately leading to the rapid clinical deterioration and death of the patient.

## 1. Introduction

According to the American Cancer Society, thyroid carcinoma represents the 13th most common malignancy worldwide and the 6th most common cancer in women, occurring approximately three times more frequently in women compared to men [1]. The majority of thyroid neoplasms are represented by differentiated thyroid carcinomas, namely papillary thyroid carcinoma (PTC) and follicular thyroid carcinoma (FTC). Other entities include medullary thyroid carcinoma and anaplastic cancer, as well as metastases to the thyroid, most of which disseminate from clear cell renal cancer or small-cell lung carcinoma [1,2].

Mucoepidermoid carcinoma (MEC) is the most common type of malignant salivary gland tumor, accounting for 10–15% of all salivary gland neoplasms, with a mean age of onset of 55 years and a slight female to male prevalence [3]. Other sites of origin for MECs include the gastrointestinal tract, the pancreas, and the breast tissue [4,5,6,7]. Rarely, primary MEC may arise from the thyroid gland, with a total of approximately 50 cases being reported to date and the disease being first described in 1977 by Ratigan et al. [8]. Histologically, primary thyroid MEC is defined by a mixture of malignant epidermal and mucin-secreting cells [9,10] which may have periodic acid–Schiff-positive intracytoplasmic inclusions, and it may be associated with chronic lymphocytic thyroiditis or concurrent papillary thyroid carcinoma [11,12].

It is postulated that thyroid MECs derive from ectopic salivary gland tissue located within the thyroid parenchyma or in the thyroid capsule itself, usually being associated with a good prognosis and indolent behavior, owing to their low-grade nature [13,14]. Another hypothesis suggests that they originate from metaplastic follicular thyroid epithelium [13,14,15]. Histologically, according to the 2022 WHO classification of thyroid tumors, MECs are a part of salivary-like carcinomas, a group which also includes sclerosing mucoepidermoid carcinoma with eosinophilia (SMECE) and secretory carcinoma (SC) [10]. Microscopically, MECs exhibit a cystic or solid growth pattern, are composed of mucinous or squamous cells arranged in nest-like or cribriform structures which contain a variable degree of sclerotic stroma, and are stain-positive for p63 and CK5 and negative for thyroglobulin and calcitonin [16,17]. In contrast with salivary MECs, keratinization is seen in as many as one third of primary thyroid MECs [18]. Although concurrent papillary thyroid carcinoma may be detected in a number of cases, the cells of the MEC component are distinct and lack the papillary-like nuclear features of the papillary component [11,12]. As opposed to salivary gland MECs, those originating in the thyroid rarely express CRCT1/MAML2 gene rearrangements, which could be a useful tool in differentiating between primary thyroid MECs and metastases to the thyroid [18,19].

As noted before, thyroid MECs usually have an indolent clinical behavior, but aggressive cases with locoregional or distant metastases have been reported [20,21,22]. First-line investigations include neck ultrasound and contrast-enhanced computerized tomography (CT) scans to establish the extent of the disease. Fine-needle aspiration biopsy (FNAB), in particular, is of little use, as cytology usually only describes atypical cells in such cases, whereas excisional or core-needle biopsies might provide sufficient material to facilitate making the correct diagnosis.

Owing to its low grade, much controversy exists in regard to its therapy. However, surgical resection based on the staging of the disease is currently the treatment of choice. Other therapeutic choices include platinum-based chemotherapy and other alkylating agents, conventional radiotherapy, and radioiodine therapy, but they have shown little benefit. More recently, HER2 overexpression has been described in MECs of salivary gland origin, which could prove useful for targeted therapy, but no such reports exist on primary thyroid MECs as of yet [23,24].

## 2. Case Report

Herein, we report the case of a 67-year-old male who presented with a four-week history of weight loss (approximately 5 kg), low-grade fever, fatigue, and dyspnea. Of note is that the patient had a known (but undocumented) history of an anterior cervical mass, diagnosed three years prior to the current presentation. He reported the recent growth of said cervical mass, but otherwise denied any other symptoms suggestive of local invasion, such as difficulty swallowing or hoarseness of the voice.

Upon physical examination, there was an enlarged, inhomogeneous, and painless thyroid gland, the left lobe of which was hard and adherent to the surrounding cervical tissues. Several hard, painless cervical adenopathies were also noted.

Blood tests showed mild normochromic anemia and inflammatory syndrome, with leucocytosis, neutrophilia, an ESR of 69 mm/h, and a CRP of 28 mg/dL. The other routine lab tests returned normal results, including those for liver enzymes and renal function. The thyroid function tests were normal, and so was calcitonin. Thyroid autoimmunity was also excluded (TPO antibody negative)

Neck ultrasound revealed an enlarged thyroid gland, along with a large hypoechoic mass in the left lobe showing microcalcifications and multiple bilateral cervical pathological lymph nodes, the biggest of which measured up to 3 cm.

**Figure 1:**
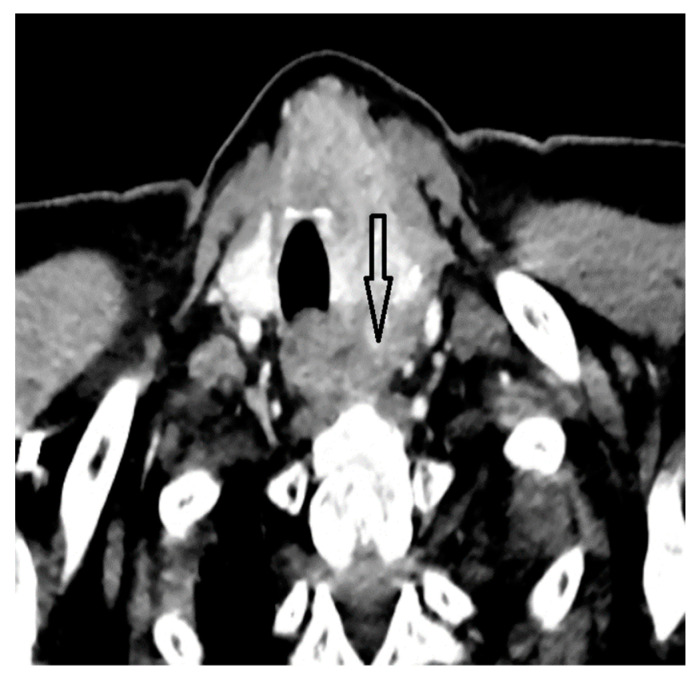
Thyroid mass comprising left lobe and isthmus.

**Figure 2:**
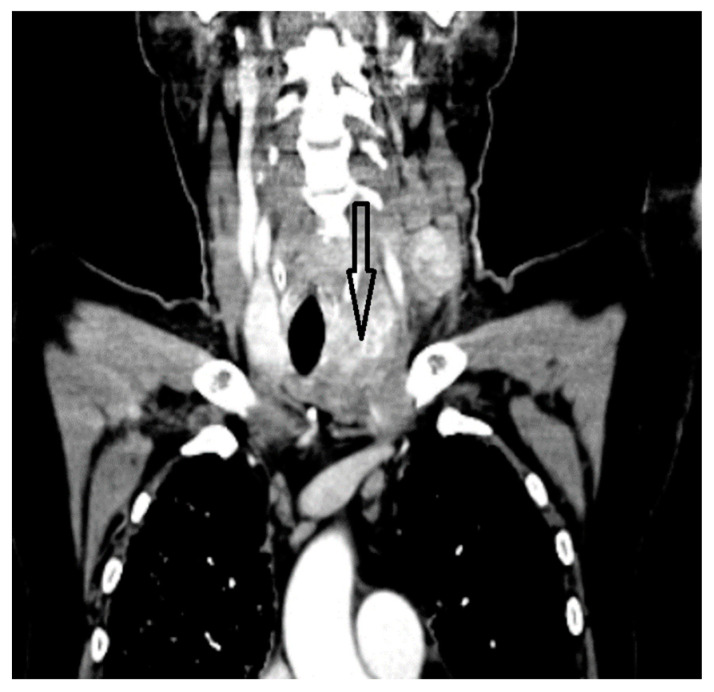
Thyroid mass with mediastinal extension.

**Figure 3:**
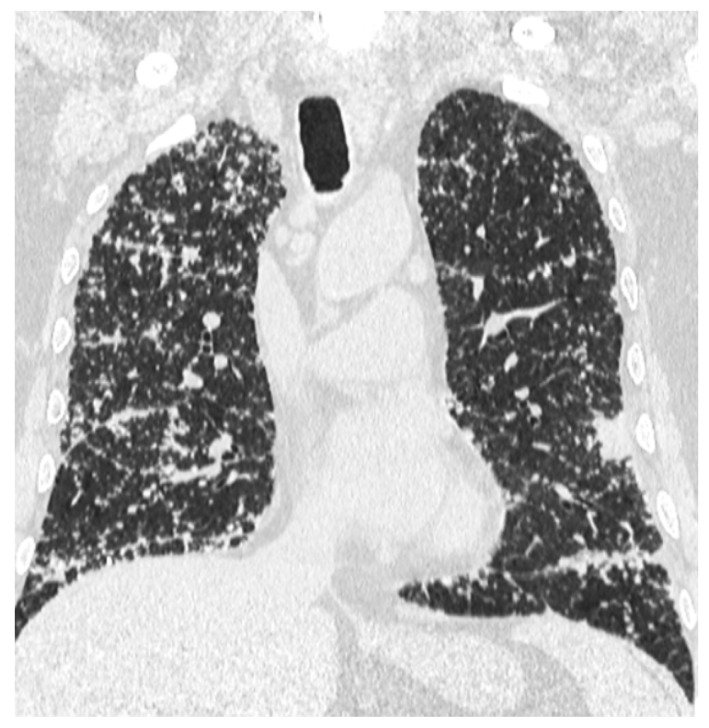
Lymphangitis carcinomatosis of the lungs.

**Figure 4:**
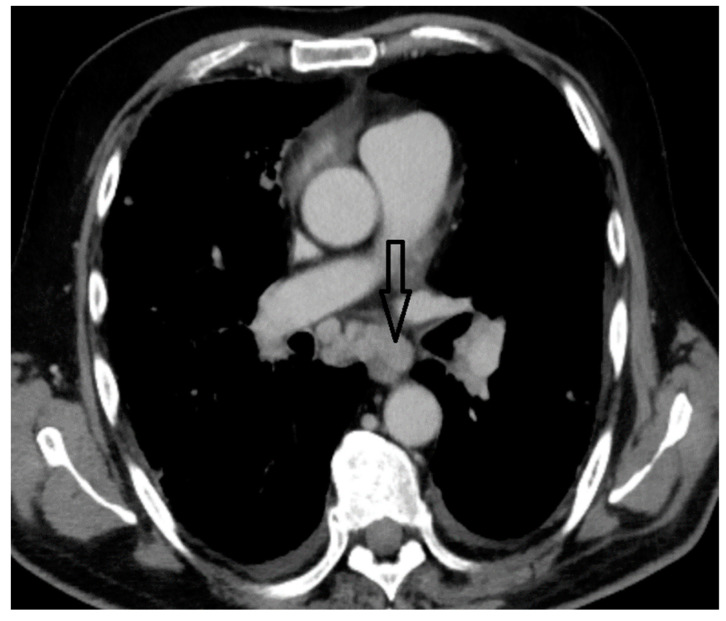
Mediastinal adenopathies.

A whole-body contrast enhanced CT scan was subsequently performed, which demonstrated a 6/2.8 cm heterogeneous cervical mass that enclosed the left thyroid lobe and isthmus, as well as the posterior side of the sternocleidomastoid muscle (with no clear demarcation line), and superior extension to the larynx and to the thyroid cartilage. Inferiorly, invasion to the superior mediastinum structures was noted. Chest CT revealed multiple bilateral pulmonary nodules, suggestive of metastasis, and lymphangitic carcinomatosis. Several cervical and mediastinal lymph nodes with a tendency to confluate were also involved (Figure 1, Figure 2, Figure 3 and Figure 4).

Following an assessment of the case as a multidisciplinary team, the patient was referred to the Surgery Department, where an excisional biopsy was performed from one of the left laterocervical adenopathies (a tissular fragment of 13/12/10 mm). Histological examination showed malignant infiltration with oval, round, and polygonal medium-sized cells arranged in a nest-like and islet-like growth pattern, findings consistent with metastasis. In order to establish the origin of malignancy, immunohistochemistry tests were performed, with CK19, p63, TTF1, and PAX8 being diffusely positive and thyroglobulin, Chromogranin A, Synaptophysin being negative in the tumor cells, thus proving the diagnosis of metastasis from primary mucoepidermoid carcinoma of the thyroid. CD5 staining was also performed in order to exclude primary metastatic thyroid lymphoma, and it was negative. Ki67 was positive in approximately 15% of tumoral cells.

**Figure 5:**
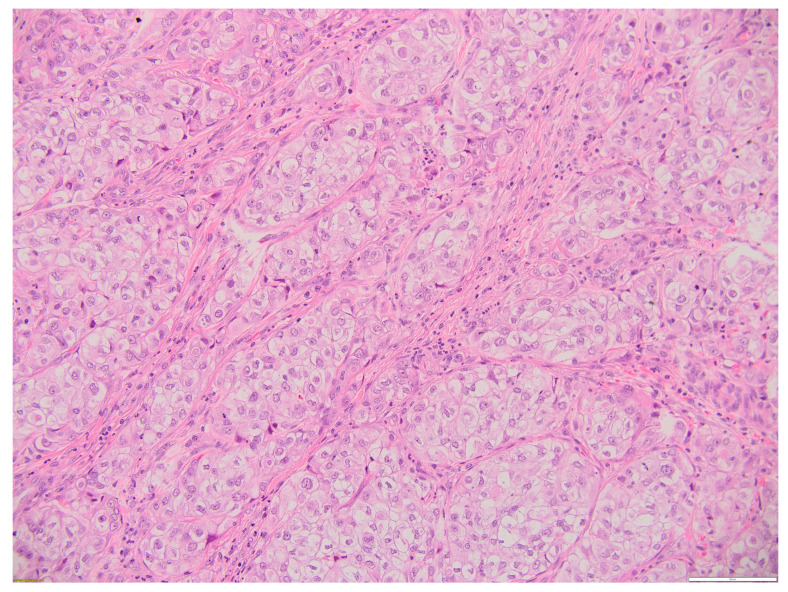
Thyroid MEC metastasis HE 20×. Oval, round, and polygonal cells arranged in a nest-like and islet-like growth pattern. Scale bar 100 µm.

**Figure 6:**
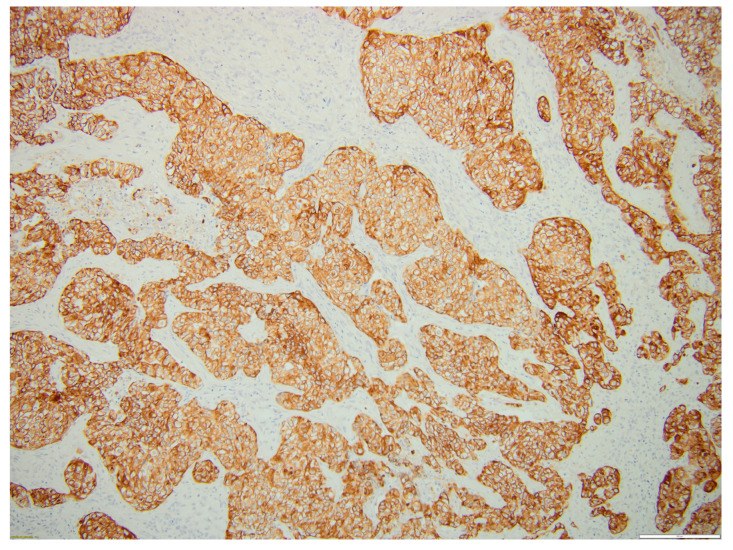
CK19-positive thyroid MEC metastasis. Scale bar 200 µm.

**Figure 7:**
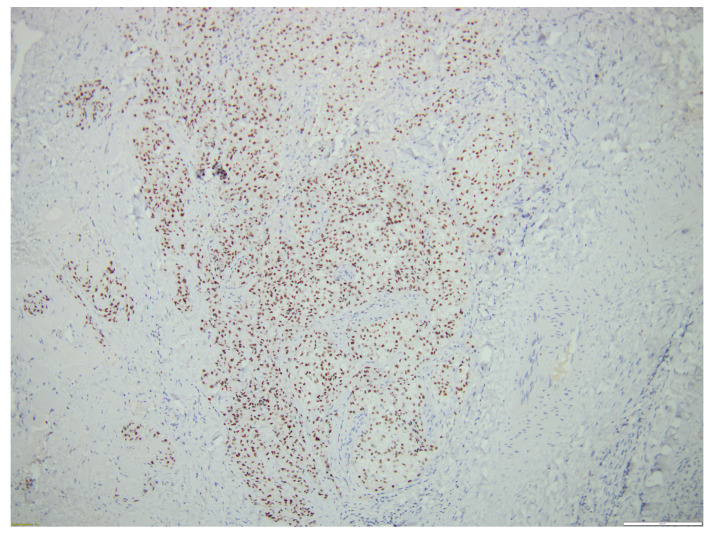
PAX8-positive thyroid MEC metastasis. Scale bar 200 µm.

**Figure 8:**
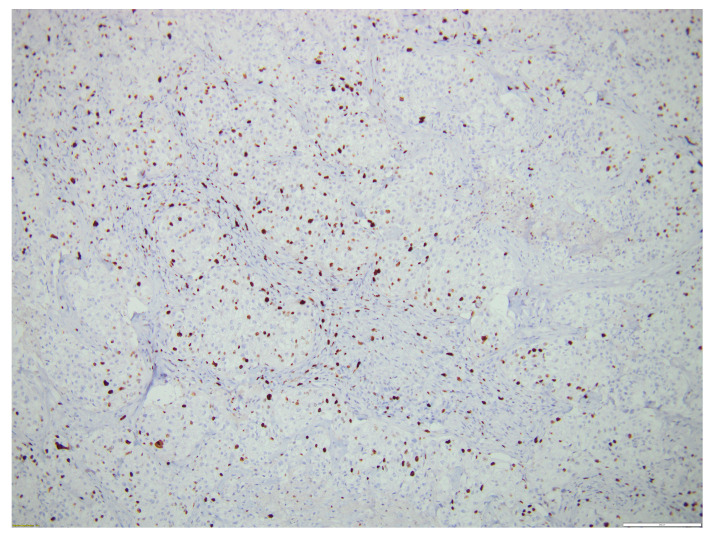
Thyroid MEC metastasis ki67+ in 15% of tumoral cells. Scale bar 200 µm.

Figure 5, Figure 6, Figure 7 and Figure 8 illustrate several histological and immunohistochemistry aspects of the tumor.

The patient was considered for total thyroidectomy and debulking surgery, but due to the worsening of the upper respiratory symptoms, the patient was referred to the Pneumology Department. Further clinical deterioration unfortunately prevented the patient from undergoing active oncological treatment, and thus the decision was made for palliative care.

While waiting for the final immuno-histopathological results and for the multidisciplinary team’s decision on the most appropriate treatment for his condition, at his request, the patient was discharged from our hospital and went home. However, in a matter of days, his status significantly deteriorated due to dyspnea, heavy breathing, severe fatigability, and desaturation (home pulse oximetry assessment showed O_2_ saturation of 80–85%). The patient was subsequently admitted to a Pneumology Department from his native town, where he unfortunately passed away in less than 24 h due to acute hypoxic respiratory failure.

## 3. Discussion

While being one of the most common malignant tumors derived from salivary gland tissue, primary thyroid MEC is an extremely rare and often poorly understood tumor, accounting for less than 0.5% of primary thyroid neoplasms [1,2]. Approximately 60 cases have been reported [25] since the first primary thyroid MEC patient was presented by Rhatigan in 1978 [8].

Mucoepidermoid carcinomas, together with sclerosing mucoepidermoid carcinoma with eosinophilia (SMECE) and secretory carcinoma, comprise a group of rare thyroid neoplasms called salivary-like carcinomas, as they histologically resemble primary salivary gland tumors. Due to their rare nature, little is known about the tumorigenesis of thyroid MECs. However, several hypotheses exist on the oncogenesis of salivary MECs, and these hypotheses could be extrapolated to their thyroid counterparts. Their common link involves the pluripotent nature of the basal cell layer of the salivary excretory duct, which may aberrantly differentiate into squamous or mucinous cells [3,13].

The microscopic findings related to thyroid MECs consist in epidermoid and mucous cells arranged in cords, nests, or solid sheets in the fibrotic stroma. Epidermoid cells have intercellular bridges and undergo keratinization, while the mucous cells have clear or vacuolated cytoplasm and exhibit a peripherally displaced nucleus. Upon immunohistochemical staining, useful markers for MECs are p63, p40, and cytokeratin 5/6 [26]

SMECE, on the other hand, was initially considered a subtype of MEC, first being reported in 1991 [27], but it is presently acknowledged as a separate entity [16]. Its clinical presentation resembles that of MECs, and it may also co-exist with chronic lymphocytic thyroiditis, but it is rarely associated with PTC compared to MEC [28,29]. The histological hallmark of SMECE is the presence of sclerotic stroma, with a variable degree of infiltration with eosinophils and other immune cells such as lymphocytes and plasma cells [30,31]. Treatment generally involves total thyroidectomy and cervical lymph node dissection in locally advanced disease. Similar to the management of MECs, conventional chemotherapy, as well as external beam radiation and radioactive iodine therapy, has been employed in the treatment of SMECE, but with little success [32,33,34]

Finally, secretory carcinoma (SC) is another type of salivary-like carcinoma of the thyroid, and it was first described in 2010 as a type of neoplasm occurring in salivary glands and resembling breast secretory carcinoma [35]. Since then, it has also been described in the skin, lung, and thyroid [36,37,38]. Compared to the other two subtypes, SC appears to be more aggressive, being usually diagnosed in older individuals and in a more advanced stage [38,39]. Histologically, they are composed of polygonal eosinophilic cells which exhibit a cribriform, trabecular, or papillary-like growth pattern [39,40]. Specifically, the malignant cells stain positive for mammary cell markers such as mammaglobin [41]. The treatment strategies resemble that of MEC and SMECE, and the majority of patients undergo total thyroidectomy as a first-line therapy. Conventional chemotherapy, radiotherapy, and radioiodine therapy have proven to be ineffective. Targeted therapy with tyrosine-kinase inhibitors has been used, albeit only in the treatment of salivary gland SC and not in primary thyroid SC [42,43].

While being a cornerstone in the diagnosis of differentiated thyroid cancers such as papillary or follicular thyroid carcinomas, neck ultrasound and FNAB are of little help in the diagnosis of MEC, often failing to distinguish between it and other rare types of thyroid neoplasms, such as anaplastic carcinoma, lymphoma, or metastases. Even in patients with classical salivary MEC, where the degree is suspicion is certainly higher, a specific diagnosis was obtained by FNA in only 39% of patients in [44]. On the other hand, core needle or excisional biopsies help provide an adequate specimen in order to confirm the diagnosis, and of particular use are IHC stainings, which are positive for p63 [45,46]. In our case, we did not perform FNA because the CT images of our patient, showing extensive nodular involvement and pulmonary metastasis, were clearly suggestive of an aggressive form of cancer, presumably non-papillary, and we considered an urgent histological examination to be needed. For practical reasons, the multidisciplinary team (endocrinologist, oncologist, and surgeon) considered that the best option would be a biopsy from the most accessible latero-cervical lymph node.

Generally, MEC arising in the thyroid is associated with an indolent clinical evolution and a favorable prognosis and usually presents as a solitary thyroid nodule, prompting evaluation [47,48]. A recent literature review on this topic [25] showed that thyroid MECs typically occur in elderly patients, mostly in men. Chronic lymphocytic thyroiditis was present in 42% of cases, while concurrent thyroid neoplasms were documented in more than 50%. About half of the patients with thyroid MECs had lymph note metastasis, but distance metastases were rare, being observed in only 15% of patients. The mean Ki67 labeling index was 14% (range: 2–40%), and 5-year survival in patients with thyroid MECs was 69.1%, confirming once more their low to intermediate aggressivity.

Nonetheless, cases of thyroid MEC behaving in an unexpectedly aggressive fashion have been described, presenting either as metastatic cervical adenopathy with local and regional invasion or distant metastasis to the lung or bone [20,21,22]. Such cases offer a poor prognosis, with survival often being limited to less than 6 months, as was the case with our patient. At diagnosis, our patient already had a very large cervical tumor, with extrathyroid invasion, as well pathologic cervical and mediastinal lymph nodes, multiple bilateral pulmonary nodules, and lymphangitic carcinomatosis. The clinical evolution was determined by the extent of the pulmonary involvement, with the patient rapidly developing acute respiratory insufficiency and exitus in less than 4 weeks from the initial presentation. A similar evolution with distant metastasis or extrathyroidal extension from the beginning was described in ten of the cases reported in the medical literature, all of whom died within the first year after diagnosis [21]. Two of these patients had lung metastasis [20,49]; however, to our knowledge, this is the first case of a patient with pulmonary lymphangitic carcinomatosis due to mucoepidermoid carcinoma of the thyroid.

In our case, the diagnosis of thyroid mucoepidermoid carcinoma was established from an analysis of one of the cervical adenopathies. At the request of the patient’s family, there were no post-mortem pathological studies, so we do not have a histological report of the thyroid tumor per se. Consequently, we do not know whether a concurrent differentiated thyroid neoplasm was present or not, as was the case in more than half of the patients reported with aggressive forms of thyroid MEC [22]

Treatment plans in the context of thyroid MECs unequivocally revolve around the surgical resection of the tumor, based on the extent of the disease. Prophylactic cervical lymph node dissection is not currently recommended, but it is commonly indicated in metastatic disease. However, there is little consensus when it comes to advanced or metastatic disease. Strategies employing the use of conventional chemotherapy and external beam radiation therapy have been used, but more often than not, these options have had limited benefits in regard to survival [20,21,22]. Radioiodine therapy has also been suggested, although MECs do not typically take up radioactive iodine [33].

Recent molecular studies have described the overexpression of HER2 and EGFR in primary salivary gland MECs [23,24]. To our knowledge, these findings have not yet been described in thyroid MECs, but more research is needed, as such receptors may prove to be the gate towards targeted therapy, especially in advanced disease.

## 4. Conclusions

Primary mucoepidermoid carcinomas of the thyroid are extremely rare tumors that are usually associated with an indolent clinical evolution and a favorable prognosis. However, there are reports of patients with primary thyroid MECs harboring more aggressive behavior, with distant metastasis from diagnosis, rapidly progressing symptoms, and limited survival. This case is an example of such an invasive and aggressive tumor, with substantial local extension and pulmonary lymphangitic carcinomatosis, along with a rapidly fatal outcome.

## Data Availability

Data are contained within the article.
